# Effects of Short-Term Treatment of Rabbit Extraocular Muscle With Ciliary Neurotrophic Factor

**DOI:** 10.1167/iovs.65.11.41

**Published:** 2024-09-27

**Authors:** Jolene C. Rudell, Linda K. McLoon

**Affiliations:** 1 Department of Ophthalmology, University of California San Diego, La Jolla, California, United States; 2 Departments of Ophthalmology and Visual Neurosciences, University of Minnesota, Minneapolis, Minnesota, United States; 3 Stem Cell Institute, University of Minnesota, Minneapolis, Minnesota, United States

**Keywords:** ciliary neurotrophic factor (CNTF), myogenic growth factors, extraocular muscles, strabismus

## Abstract

**Purpose:**

Little is known about the effect of ciliary neurotrophic factor (CNTF) on extraocular muscles, but microarray studies suggested CNTF might play a role in the development and/or maintenance of strabismus. The effect of short-term treatment of adult rabbit extraocular muscle with injected CNTF was examined for its ability to alter muscle characteristics.

**Methods:**

Eight adult New Zealand white rabbits received an injection into one superior rectus muscle of 2 µg/100 µL CNTF on 3 consecutive days. One week after the first injection, the rabbits were euthanized, and the treated and contralateral superior rectus muscles were assessed for force generation capacity and contraction characteristics using an in vitro stimulation protocol and compared to naïve control superior rectus muscles. All muscles were analyzed to determine mean cross-sectional areas and expression of slow twitch myosin heavy chain isoform.

**Results:**

Short-term treatment of rabbit superior rectus muscles with CNTF resulted in a significant decrease in muscle force generation, but only at the higher stimulation frequencies. Significantly decreased myofiber cross-sectional areas of the treated muscles correlated with the decreased generated force. In addition, there were significant changes to contractile properties of the treated muscles, as well as a decrease in the number of myofibers expressing slow twitch myosin heavy chain.

**Conclusions:**

We show that short-term treatment of a single rabbit superior rectus muscle results in decreased myofiber size, decreased force, and altered contractile characteristics. Further studies are needed to determine if it can play a role in improving alignment in animal models of strabismus.

There is a great deal of evidence that neurotrophic factors play a significant role in development and maintenance of skeletal muscles and their innervating motor neurons generally, but they are very important in extraocular muscles and their innervating ocular motor neurons specifically.^1^^,^^2^ The extraocular muscles and their innervating motor neurons express higher levels of neurotrophins than limb skeletal muscles,^3^^,^^4^ and these are retained at high levels in motor neuron diseases where the extraocular muscles are preferentially spared from degeneration.^5^^–^^7^ These data suggested that these neurotrophic and growth factors play roles in both development and maintenance of the specific properties of ocular motor function.

Studies also have demonstrated the ability of exogenously added growth factors and neurotrophic factors to modulate extraocular muscle structure and function significantly.^8^ For example, insulin-like growth factor I (IGFI) resulted in a significant increase in muscle size and force generation in both adult rabbit and chick extraocular muscles,^9^^,^^10^ while sustained treatment with fibroblast growth factor 2 (FGF2) in rabbits resulted in decreased muscle size and force generation.^11^ Sustained treatment of an adult strabismic monkey with IGFI resulted in a significant improvement in eye alignment,^12^ and sustained treatment with either IGF1 or glial derived neurotrophic factor (GDNF) during the first 3 months of life in nonhuman primates resulted in development of strabismus.^13^^,^^14^ This demonstrates that sustained treatment with neurotrophic factors may be a more effective long-term treatment for individuals with strabismus that overrides the compensatory mechanisms of the visual sensorimotor system that occurs due to the sudden changes in eye alignment after strabismus surgery.^15^^,^^16^ DNA microarray studies examined extraocular muscles from children and adults with strabismus and compared them to control extraocular muscles.^17^^,^^18^ One of the genes that they found down-regulated was ciliary neurotrophic factor (CNTF). As the muscles used for analysis were from individuals with strabismus and age-matched controls, it is unclear whether the altered levels of CNTF were primary or secondary to other changes in the neurotrophic factor milieu in individuals with strabismus. Its role in extraocular muscle or ocular motor neuronal development is unclear; however, it has been shown to play a role in supporting the survival of specific groups of motor neurons in development.^19^ Based on these studies, we set out to determine if treatment of adult rabbit extraocular muscles would result in changes to muscle force generation and myofiber size.

## Methods

New Zealand white rabbits from Bakkom Rabbitry (Viroqua, WI, USA) were housed under the care of Research Animal Resources in the AALAC-approved facilities at the University of Minnesota. All experiments were approved by the Institutional Animal Care and Use Committee, and followed the guidelines of the Association for Research in Vision and Ophthalmology and the National Institutes of Health for using animals in research. Animals were housed on a 12-hour light/dark cycle and had food ad libitum.

In preparation for muscle injections, the 8 rabbits were anesthetized with an intramuscular injection of ketamine:xylazine at a dose of 10 mg/kg:2 mg/kg. For 3 consecutive days, the superior rectus on one side received an injection of 2 µg CNTF (Peprotech. Inc., Rocky Hill, NJ, USA) in 100 µL sterile buffered saline, with the same volume of saline only injected into the contralateral superior rectus muscle. Treated muscles were selected randomly. An additional six animals served as naïve controls. On the seventh day after the first injection, the animals were deeply anesthetized, exsanguinated, and the right and left superior rectus muscles were dissected from the sclera to the apex. All animals were numbered, and we were masked as to which muscle was treated when they were tested. These were placed in oxygenated Ringer's solution and prepared for in vitro force assessment using methods standard in the laboratory for in vitro assessment of muscle force development after stimulation.^11^^,^^20^ In brief, both muscle ends were tied with sutures, and these were then attached to a calibrated lever arm and placed in a temperature-controlled bath surrounded by oxygenated Ringer's solution. After force-length tension curves were generated, the muscle was set at the appropriate length tension. The right and left muscles were tested at each stimulation sequentially, and flanking electrodes applied a stimulus that ranged from single twitch pulse with a duration of 500 msec, followed by stimulation at 10, 20, 40, 100, 150, and 200 Hz, and a 2-minute rest between each stimulus. A muscle fatigue protocol was then applied, which consisted of a 1-second train of a 150 Hz tetanic stimulus every 2 seconds for a total of 600 seconds or until there was a 50% reduction in generated muscle force. At the end of the physiology experiments, the muscles were weighed and the total muscle length was measured. Force was measured in grams and converted to mN/cm^2^ by dividing muscle mass (grams) by the product of muscle length (centimeters) times the skeletal muscle density of 1.056 g/cm^2^. This normalizes the raw force values to muscle size. Data were pooled for treated and contralateral superior rectus muscles for each of the stimulation intensities. Data from naïve control superior rectus muscles were compared to the treated and contralateral muscle forces. Statistics were performed using Graphpad Prism Software (version 10; Graphpad, San Diego, CA, USA). ANOVAs were used for analysis, followed by post hoc multiple comparisons to determine if data were statistically significant. Significant difference was defined as *P* ≤ 0.05.

The superior rectus muscles were embedded in tragacanth gum, frozen in liquid-nitrogen cooled 2-methylbutane and stored at –80^o^C until sectioned. The muscles were cryo-sectioned in a Leica cryostat at 12 µm. From each of eight rabbits, three cross-sections from both the mid-region of the muscle and toward the tendon end were stained with hematoxylin and eosin. All specimens were numbered, and all measurements were made masked to whether they were treated, contralateral to the treatment, or naïve control. Cross-sectional areas were determined from these slides by manual tracing using the Bioquant Morphometry System (Bioquant, Nashville, TN, USA). A minimum of 200 individual myofibers in both the orbital and global layers were measured from each of the three slides from the two muscle areas, and these were averaged to give the mean cross-sectional areas for each rabbit muscle.

Another set of similarly located cross-sections were immunostained for expression of slow twitch myosin heavy chain isoform (*myh7*). Briefly, the slides were rinsed in phosphate buffered saline (PBS), blocked in blocking buffer (20% goat serum and 0.2% bovine serum albumin in antibody buffer [0.1% Triton X-100 in PBS]) for 30 minutes at room temperature. This was followed by incubation for 1 hour at room temperature in antibody to the slow twitch myosin heavy chain isoform (*myh7*, 1:20; Developmental Studies Hybridoma Bank [DSHB] 4.951, Ames, IA, USA). The 4.951 monoclonal antibody was developed by H. M. Blau and was obtained from the DSHB, created by the National Institute of Child Health and Human Development (NICHD) of the National Institutes of Health (NIH), and maintained at The University of Iowa, Department of Biology, Iowa City, Iowa, USA. After a PBS rinse, the slides were again blocked with blocking buffer followed by incubation with goat-anti-mouse PLUS Alexafluor 488 (1:1000; A32723; Invitrogen, Carlsbad, CA, USA) for 1 hour at room temperature. After the rinses in PBS, the slides were coverslipped using Vectashield Vibrance anti-face mounting medium (H-1700; Vector Labs., Newark, CA, USA). They were imaged using a Leica microscope prior to analysis using Bioquant to determine percent positive compared to total fiber number. All specimens were numbered, and all measurements were made masked to whether they were treated, contralateral to the treatment, or naïve control. To perform this analysis, random areas in individual cross-sections were analyzed in both the orbital and global layers, with three slides in the mid-region and three slides toward the tendon region, as outlined above. These were averaged to determine the average percent positive per muscle region. ANOVAs were used for analysis, followed by post hoc multiple comparisons to determine if data were statistically significant. Significant difference was defined as *P* ≤ 0.05.

## Results

**Figure 1.**
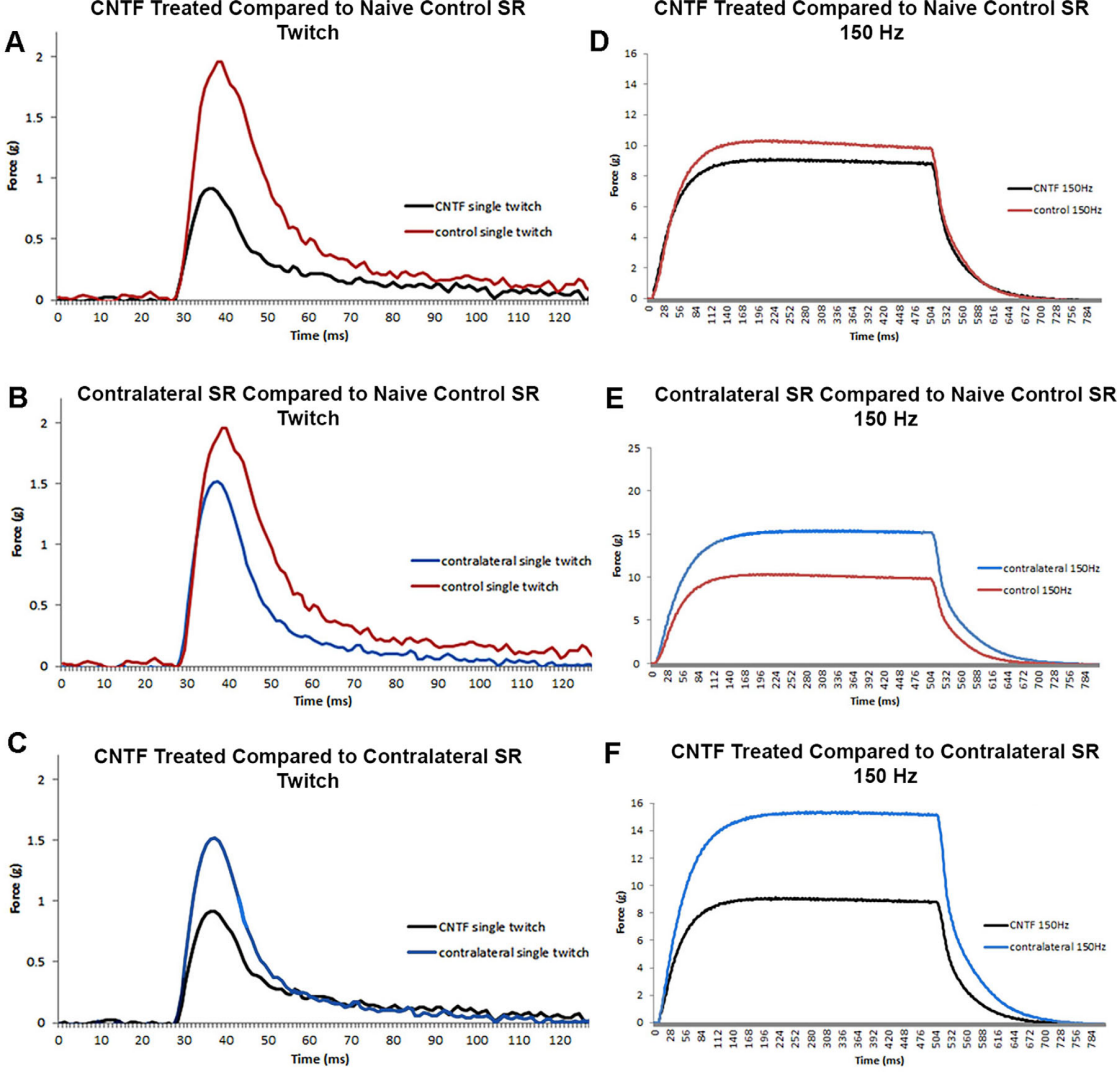
Representative traces of force in grams generated from rabbit superior rectus muscles at twitch (**A, B, C**) and 150 Hz (**D, E, F**) stimulation frequencies. Examples of (**A**) Twitch and (**D**) 150 Hz stimulation forces generated in a CNTF-treated muscle compared to a naïve control muscle. Examples of (**B**) Twitch and (**E**) 150 Hz stimulation forces generated in the muscles contralateral to the treated muscle compared to a naïve control muscle. Examples of (**C**) Twitch and (**F**) 150 Hz stimulation forces generated in a CNTF-treated muscle compared to the muscle contralateral to the treatment. CNTF-treated muscles = *black*; contralateral muscles = *blue*; and naïve control muscles = *red*.

**Figure 2.**
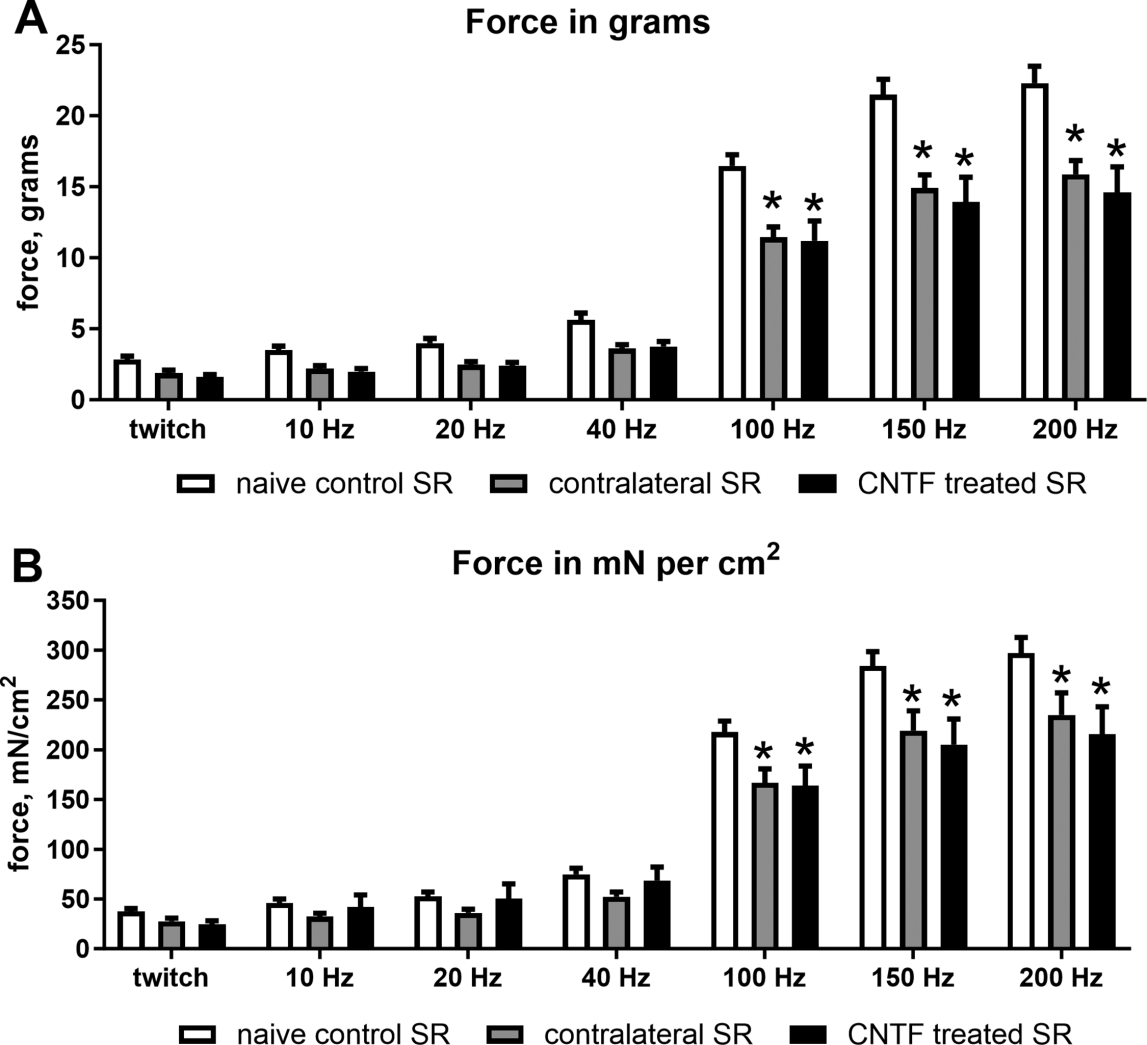
Mean force generation produced by CNTF-treated superior rectus muscles, the superior rectus muscles contralateral to treatment, and naïve control superior rectus muscles. (**A**) Force in grams at twitch, 10 Hz, 20 Hz, 40 Hz, 100 Hz, 150 Hz, and 200 Hz stimulation frequencies. (**B**) Force normalized to muscle weight at twitch, 10 Hz, 20 Hz, 40 Hz, 100 Hz, 150 Hz, and 200 Hz stimulation frequencies. There were eight CNTF-treated muscles, eight muscles contralateral to treatment, and six muscles from naïve controls. * Indicates significant difference from naïve controls at *P* < 0.05.

The short-term treatment of rabbit superior rectus muscle with CNTF resulted in a significant decrease in both muscle force in grams but only at the highest stimulation frequencies as demonstrated by ANOVA (Figs. 1, 2A). Post hoc *t*-tests demonstrated that at 100 Hz, force generation was decreased from the naïve control levels by 30.5% for the contralateral superior rectus muscle (*P* = 0.001) and 32% for the CNTF injected muscle (*P* = 0.0001), 30.6% (*P* = 0.0001), and 35.2% (*P* = 0.0001), respectively, at 150 Hz stimulation, and 28.8% and 34.6% decreases respectively at 200 Hz (*P* = 0.0001). Similarly, ANOVA showed that muscle force equalized based on muscle weight also was significantly different. Post hoc *t*-tests showed a significant decrease over the levels in naïve control muscles, again only at the higher stimulation frequencies (see Figs. 1, 2B). At 100 Hz, equalized force generation was decreased by 23.5% (*P* = 0.0001) and 24.7% (*P* = 0001) for the side contralateral to the injections and the CNTF-treated muscles respectively, at 150 Hz, it was decreased by 22.9% (*P* = 0001) and 27.8% (*P* = 0.0001), and at 200 Hz, 20.9% (*P* = 0.0001) and 27.4% (*P* = 0.0001), respectively.

**Figure 3.**
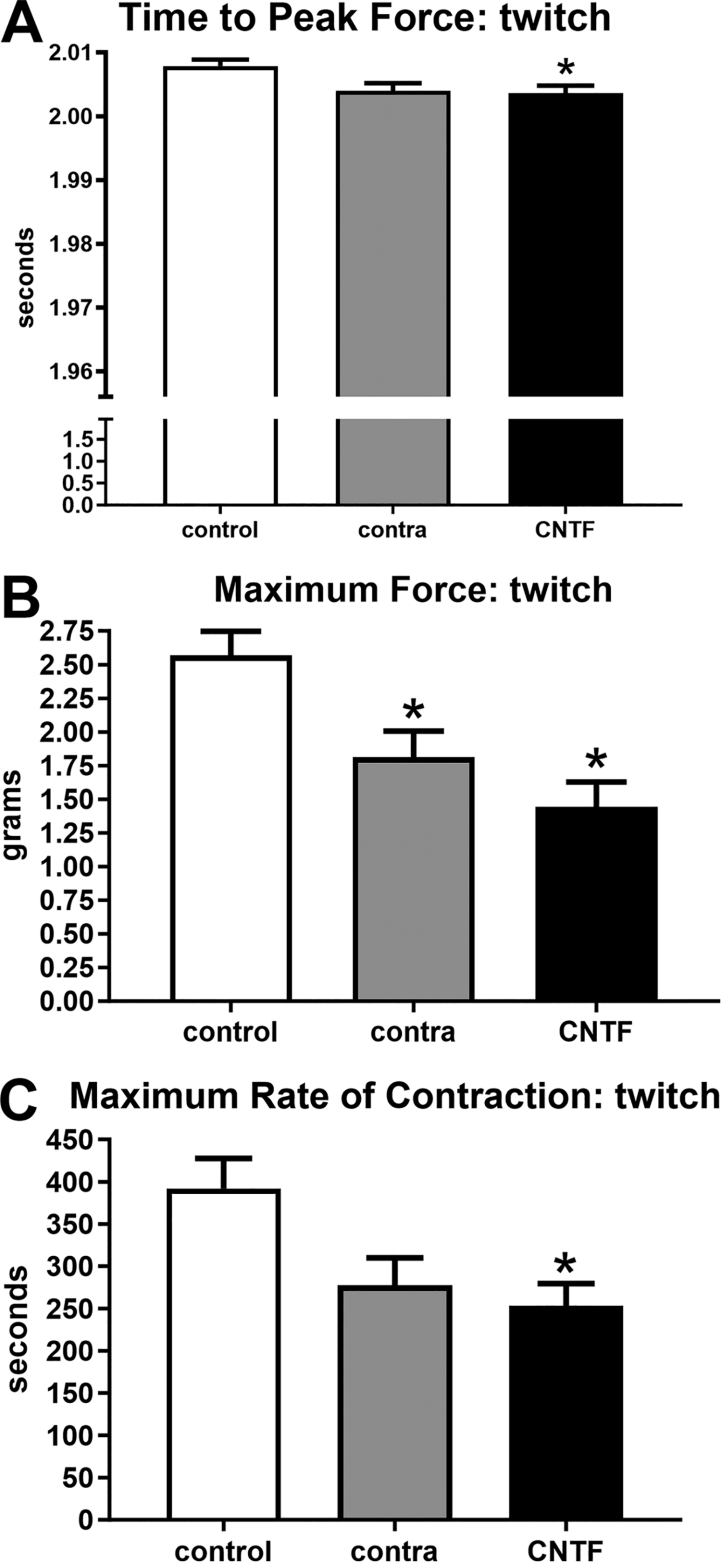
Contractile properties of superior rectus muscles were analyzed at twitch stimulation frequency. For naïve controls (control), the superior rectus muscles contralateral to the CNTF-treated muscles (contra), and the CNTF-treated muscles (CNTF), calculations were performed for (**A**) time to peak force, (**B**) maximum rate of contraction, and (**C**) maximum force. * Indicates significantly different from naïve control superior rectus muscles at *P* < 0.05.

A number of contractile characteristics significantly changed as a result of these short-term treatments with CNTF, as demonstrated by ANOVA. When examining twitch contractile characteristics using post hoc *t*-tests, the time to peak force was shorter for the CNTF-treated muscles compared to the naïve control muscles (*P* = 0.03; Fig. 3). There was no significant difference between the CNTF-treated muscles and the muscles contralateral to the treatment. Similarly, the maximal rate of twitch contraction was significantly faster after CNTF treatment (35.4%, *P* = 0.018; see Fig. 3). Although the muscle contralateral to the CNTF treatment showed a 29.3% faster response, this was not significantly different from the naïve control muscles (*P* = 0.057). There was no significant difference between the CNTF-treated muscles and the muscles contralateral to the treatment. When maximum twitch force was compared (see Fig. 3), both the CNTF-treated and the muscles contralateral to the treatment generated significantly less force than the naïve control superior rectus muscles (CNTF-treated = 43.9%, *P* = 0.0007; contralateral to treatment = 29.4%, *P* = 0.213, respectively). There was no statistically significant difference between the CNTF-treated muscles and the side contralateral to treatment.

**Figure 4.**
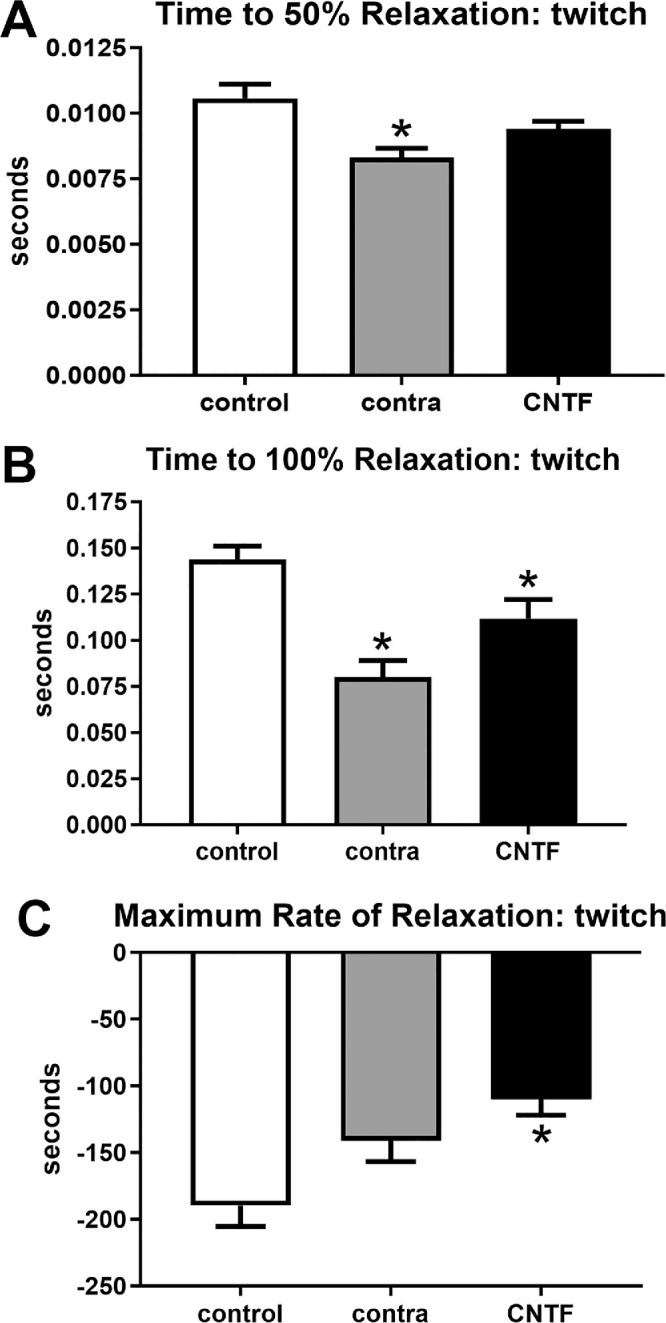
Contractile properties of superior rectus muscles were analyzed at twitch stimulation frequency. For naïve controls (control), the superior rectus muscles contralateral to the CNTF-treated muscles (contra), and the CNTF-treated muscles (CNTF), calculations were performed for (**A**) time to 50% relaxation, (**B**) time to 100% relaxation, and (**C**) maximum rate of relaxation. * Indicates significantly different from naïve control superior rectus muscles at *P* < 0.05.

We also examined relaxation parameters after twitch stimulation in these muscles, and ANOVA analysis demonstrated these to be significantly different (Fig. 4). Post hoc *t*-tests showed that time to 50% relaxation was significantly faster in the superior rectus muscle contralateral to the CNTF injections (27.5%, *P* = 0.002); however, the rate to 50% relaxation was not significantly different in the CNTF-treated muscles compared to the naïve controls (*P* = 0.139). There was no significant difference between the CNTF-treated muscles and the muscles contralateral to the treatment. When time to 100% relaxation was examined after twitch stimulations, the times were significantly reduced in both the CNTF-treated and the muscle contralateral to the CNTF treatment compared to the naïve control muscles (22.2%, *P* = 0.03; and 44.3%, *P* = 0.0001). There was no significance difference between the CNTF-treated muscles and the muscles contralateral to the treatment, although it was trending toward significance (*P* = 0.0527). As would be predicted, the maximum rate of relaxation after twitch stimulation was faster after the CNTF treatment (41.9%, *P* = 0.0025). Although the rate in the superior rectus muscles contralateral to the CNTF treatment was 25.6% faster than that of the naïve controls, it was not significantly different (*P* = 0.074). In addition, there was no significant difference between the CNTF-treated muscles and the muscle contralateral to the treatment.

**Figure 5.**
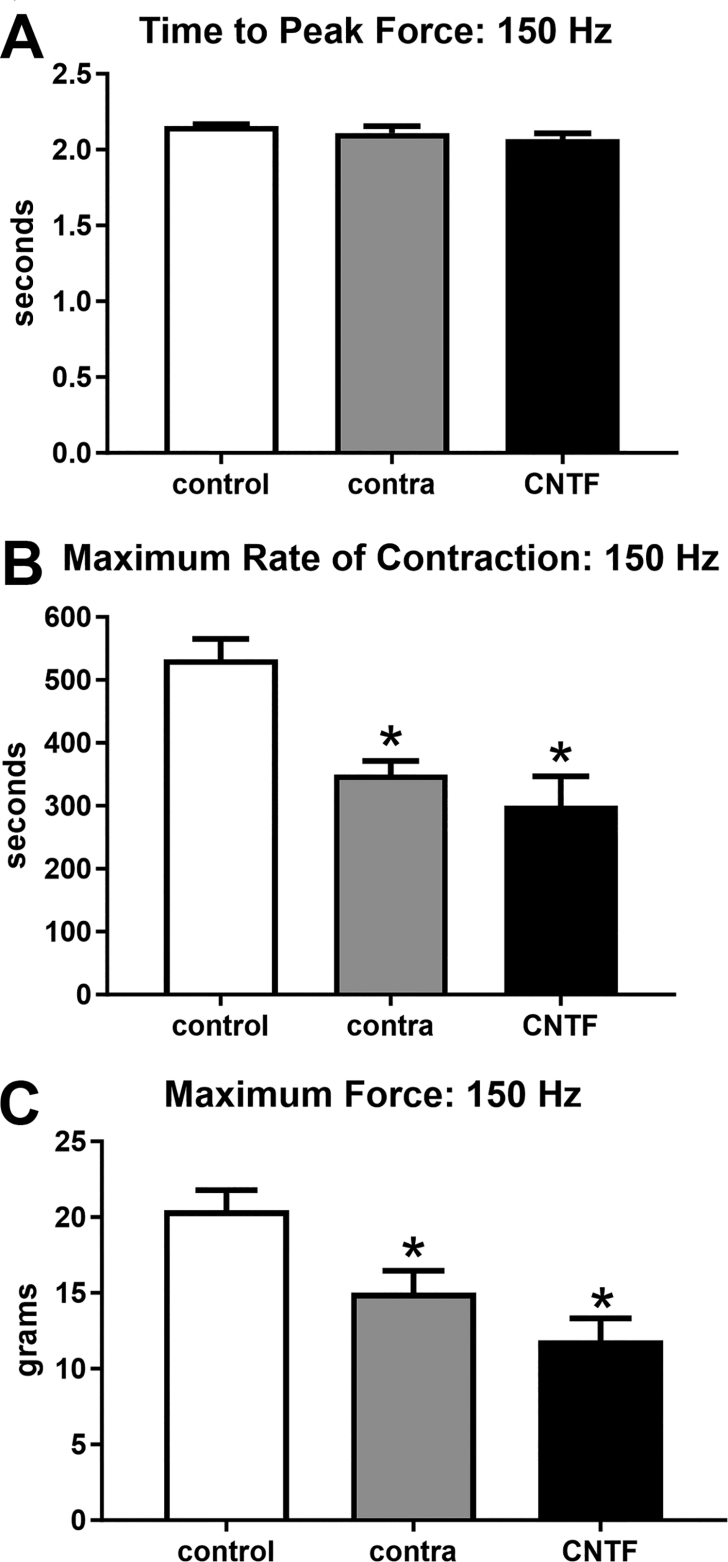
Contractile properties of superior rectus muscles were analyzed at 150 Hz stimulation frequency. For naïve controls (control), the superior rectus muscles contralateral to the CNTF-treated muscles (contra), and the CNTF-treated muscles (CNTF), calculations were performed for (**A**) time to peak force, (**B**) maximum rate of contraction, and (**C**) maximum force. * Indicates significantly different from naïve control superior rectus muscles at *P* < 0.05.

**Figure 6.**
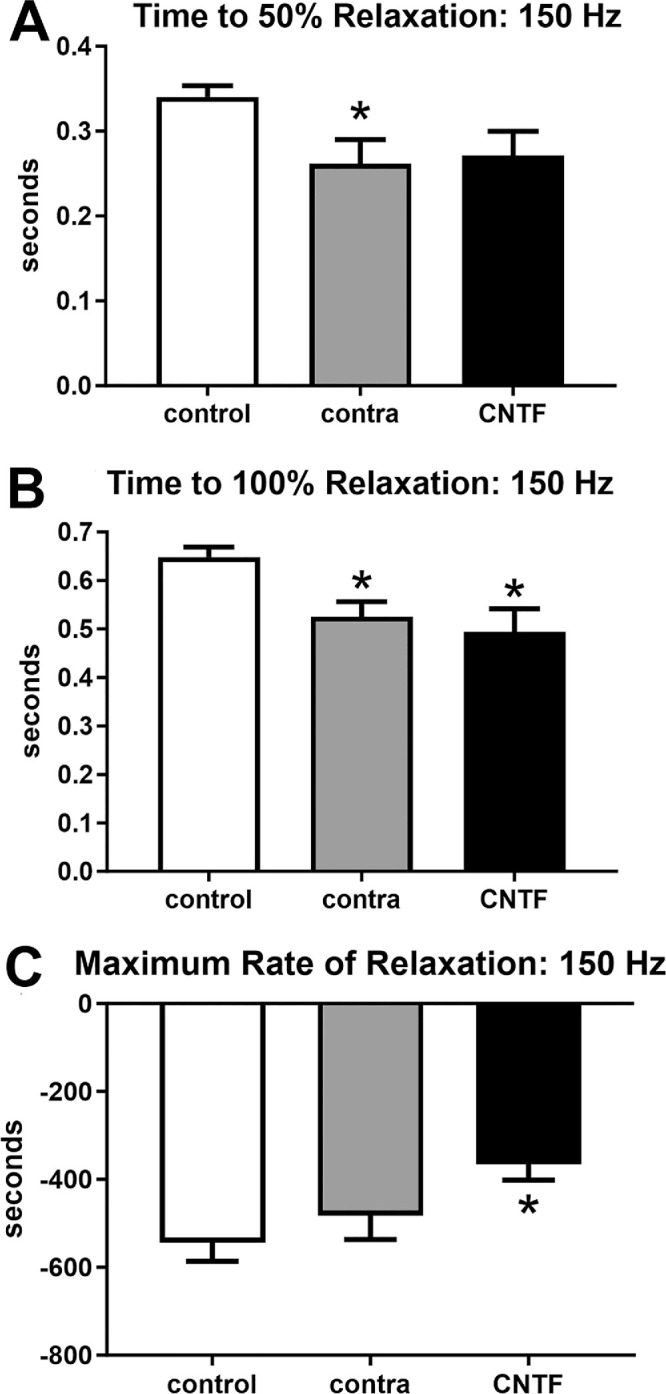
Contractile properties of superior rectus muscles were analyzed at 150 Hz stimulation frequency. For naïve controls (control), the superior rectus muscles contralateral to the CNTF-treated muscles (contra), and the CNTF-treated muscles (CNTF), calculations were performed for (**A**) time to 50% relaxation, (**B**) time to 100% relaxation, and (**C**) maximum rate of relaxation. * Indicates significantly different from naïve control superior rectus muscles at *P* < 0.05.

These same 3 sets of contractile characteristics were examined at 150 hertz (Hz) stimulation, where ANOVAs showed them to be significantly different (Figs. 5, 6). However, post hoc *t*-tests showed that after 150 Hz stimulation, there were no significant differences compared to the naïve controls in time to peak force (see Fig. 5). At 150 Hz stimulation, the maximum rate of contraction was significantly faster for both the CNTF-treated superior rectus muscle and those contralateral to the treatment compared to the naïve control muscles (CNTF-treated = 43.63%, *P* = 0.0004; muscles contralateral to the treatment = 34.4%, *P* = 0.004; see Fig. 5). There was no significant difference between the CNTF-treated muscles and the muscles contralateral to the treatment. Similarly, at 150 Hz stimulation, maximum force was significantly decreased both in the superior rectus muscles after CNTF treatment and in the superior rectus muscles on the contralateral side compared to the forces generated in naïve control muscles (CNTF-treatment = 42.0%, *P* = 0.001; muscles contralateral to the treatment = 26.7%, *P* = 0.03; see Fig. 5). ANOVAs showed that all three physiological measures of relaxation parameters were significantly different. Post hoc *t*-tests showed that the time to 50% relaxation after 150 Hz stimulation was significantly faster in the muscles contralateral to the CNTF treatment (23.1%, *P* = 0.03) compared to naïve control muscles. In the CNTF-treated muscles, despite the 20.4% faster rate of 50% relaxation, this was not significantly different from the naïve controls (*P* = 0.06; see Fig. 6). There was no significant difference between the CNTF-treated muscles and the muscles contralateral to the treatment. Times to 100% relaxation after 150 Hz stimulation were significantly faster in both the superior rectus muscles treated with CNTF and those contralateral to the treatment than the naïve controls (CNTF-treatment = 23.93%, *P* = 0.007; muscles contralateral to the CNTF treatment = 19%, *P* = 0.03; see Fig. 6). There were no significant differences between the CNTF-treated muscles and the muscles contralateral to the treatment. The maximum rates of relaxation were significantly faster in the CNTF treated muscles compared to the naïve controls (32.7%, *P* = 0.003); however, the maximum rate of relaxation was not significantly faster in the muscles contralateral to the treatment compared to the naïve controls (11.3%, *P* = 0.07; see Fig. 6). There were no significant differences between the CNTF-treated muscles and the muscles contralateral to the treatment.

**Figure 7.**
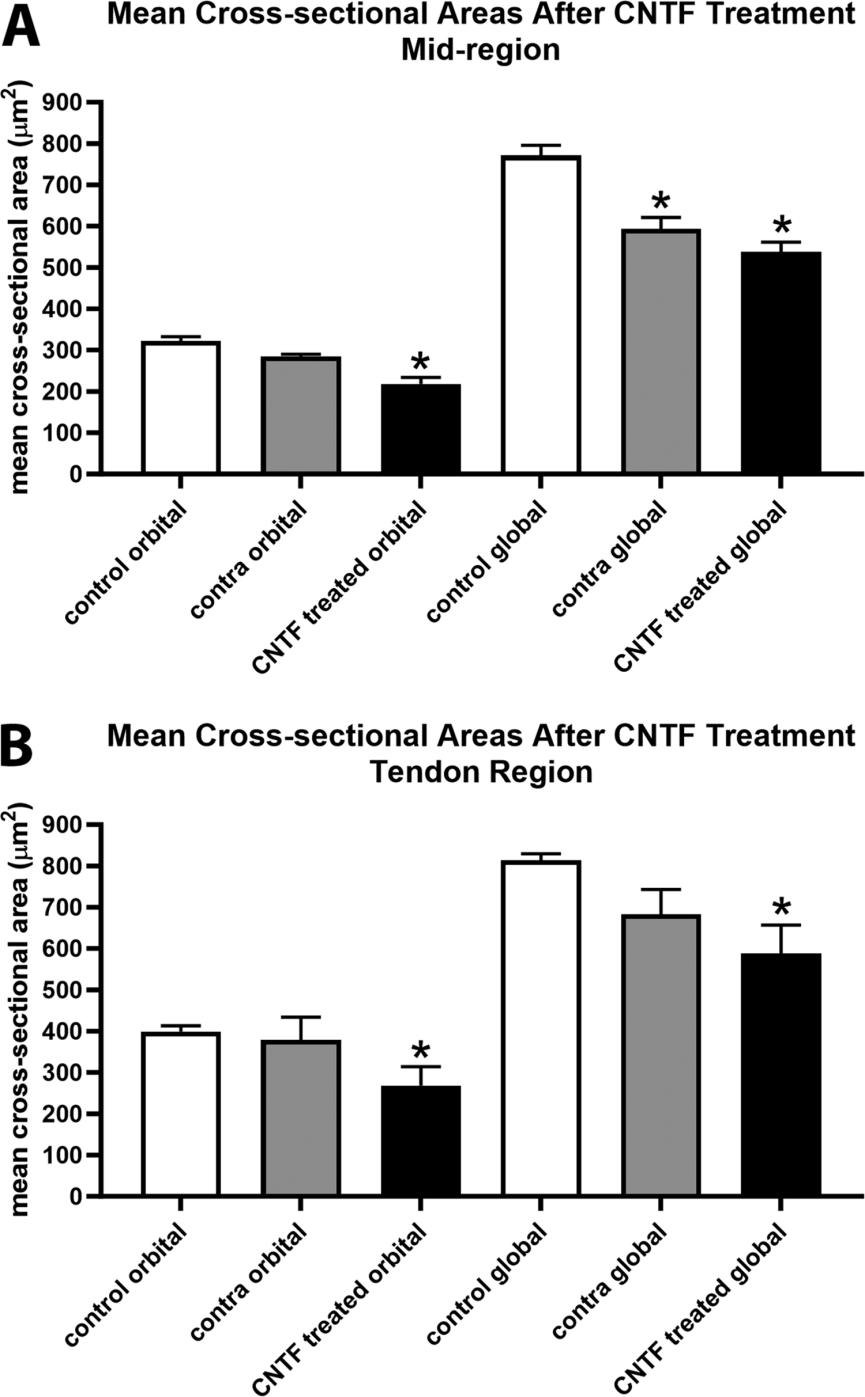
Mean myofiber cross-sectional areas were determined for naïve controls (control), the superior rectus muscles contralateral to the CNTF-treated muscles (contra), and the CNTF-treated muscles (CNTF) in both the orbital and global layers in (**A**) the mid-region of the muscles and (**B**) in a region toward the tendon end. * Indicates significantly different from naïve control superior rectus muscles at *P* < 0.05. There were eight CNTF-treated muscles, eight muscles contralateral to treatment, and six muscles from naïve controls.

The superior rectus muscles were assessed for alterations in mean cross-sectional areas as a result of CNTF treatment in the mid-region of the muscles and toward the tendon end (Fig. 7), and ANOVA showed them to be significantly different. Post hoc *t*-tests showed that in the mid-region of the superior rectus muscles, in the orbital layer the mean cross-sectional areas of the CNTF-treated superior rectus muscles were significantly decreased from that of naïve controls (32.2%, *P* = 0.001; see Fig. 7A). The orbital layer muscle fibers contralateral to the treatment were not significantly different from the naïve controls (*P* = 0.91), nor was there a significant difference between the CNTF-treated muscles and the orbital layer fibers contralateral to the treatment (*P* = 0.14). In the global layer, post hoc *t*-tests showed that in the mid-region of the superior rectus muscles the mean cross-sectional areas of the CNTF-treated superior rectus muscles were significantly decreased from that of naïve control (30.3%, *P* = 0.0001). Only in the global layer were the mean cross-sectional areas of the contralateral superior rectus muscles significantly smaller than those in the naïve controls (23.1%, *P* = 0.001; see Fig. 7A). There was no significant difference between the CNTF-treated muscles and the muscles contralateral to treatment. In the area closer to the tendon region, post hoc *t*-tests demonstrated that in both the orbital and global layers, the mean cross-sectional areas of the CNTF-treated superior rectus muscles were significantly decreased from that of naïve controls (orbital = 32.8%, *P* = 0.04; and global = 27.8%, *P* = 0.009). There were no significant differences between the mean areas for the muscles contralateral to the treatment compared to the naïve controls in either the orbital or global layers. There were also no significant differences between the CNTF-treated orbital or global muscle fibers compared to those on the side contralateral to the treatment.

**Figure 8.**
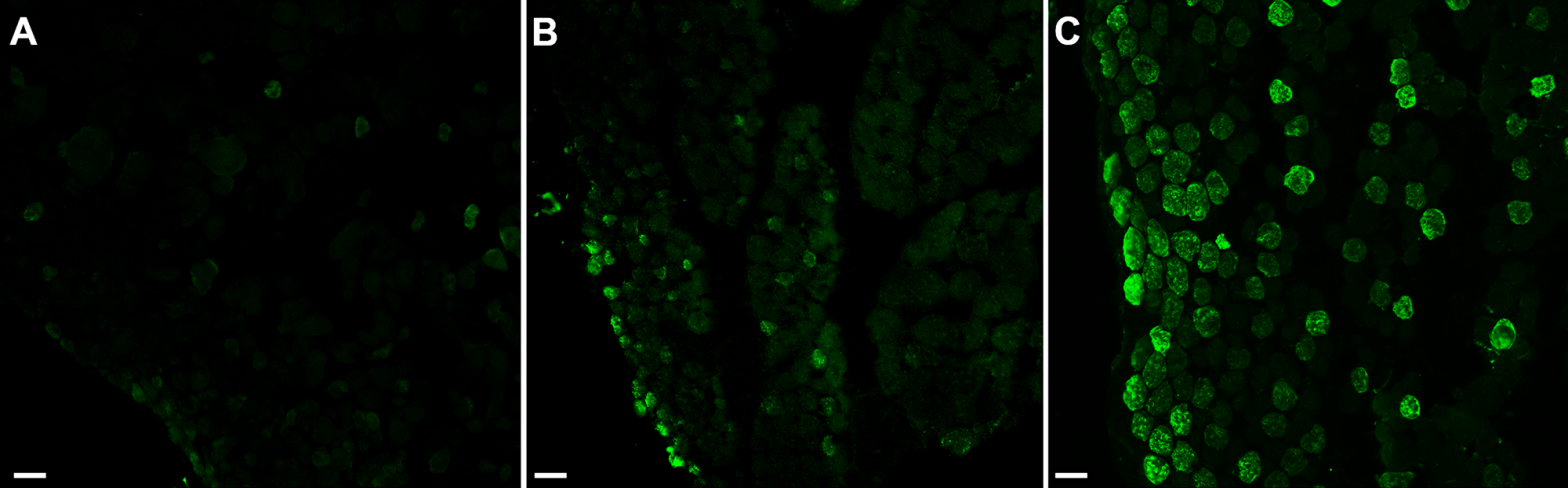
Immunostained sections with slow twitch myosin heavy chain of rabbit superior rectus muscles from (**A**) CNTF-treated muscle, (**B**) muscle contralateral to the treated muscle, and (**C**) naïve control muscle. Bar is 30 µm.

**Figure 9.**
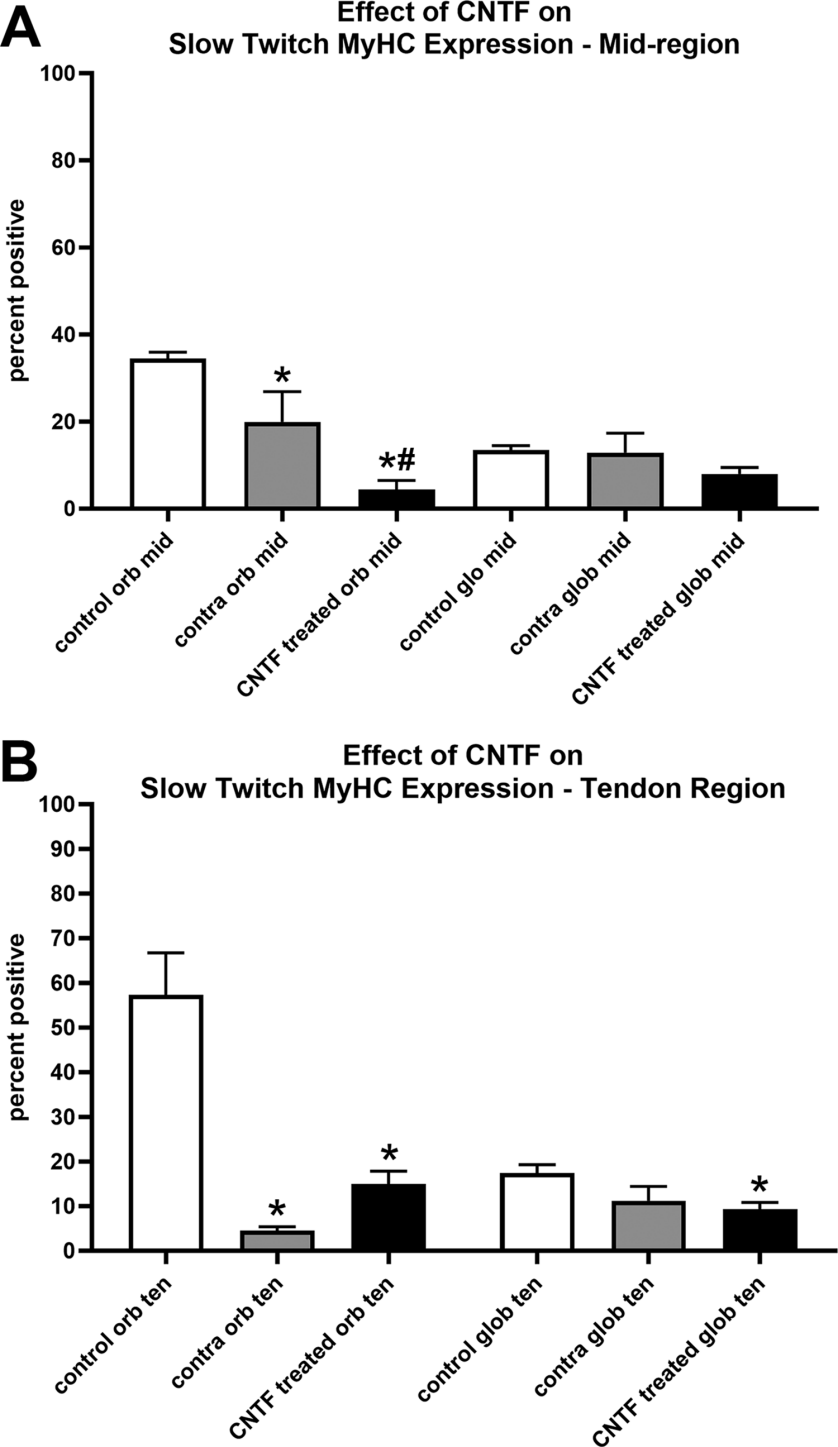
Percent of myofibers expressing the slow twitch myosin heavy chain isoform (MyHC) was determined for naïve controls (control), the superior rectus muscles contralateral to the CNTF-treated muscles (contra), and the CNTF-treated muscles (CNTF) in both the orbital and global layers in (**A**) the mid-region of the muscles and (**B**) in a region toward the tendon end. There were eight CNTF-treated muscles, eight muscles contralateral to treatment, and six muscles from naïve controls. * Indicates significantly different from naïve control superior rectus muscles at *P* < 0.05. # Indicates significantly different from the muscles contralateral to the treatment at *P* < 0.05.

Decreased force generation but increased contractile and relaxation velocities suggested there may be an alteration in slow twitch myosin heavy chain (MyHC) isoform expression patterns. ANOVA showed the orbital layer muscles to be significantly different from the naïve controls. Post hoc *t*-tests showed that compared to the naïve control muscles, there was a loss of slow twitch MyHC expression in the orbital layers of both the mid-region and tendon region of both the CNTF-treated superior rectus muscles (mid-region = 87.2%, *P* = 0.0001; tendon region = 74.0%, *P* = 0.0028) and the superior rectus muscles contralateral to the treatment (mid-region = 42.3%, *P* = 0.017; tendon region = 92%, *P* = 0.0008; Figs. 8, Fig. 9). There was also a significant difference in the mid-region between the CNTF-treated muscles in the orbital region compared to the muscles contralateral to the treatment (*P* = 0.02). In the global layer, the only significant difference was seen in the tendon region of superior rectus muscles treated with CNTF compared to the naïve control muscle fibers (tendon region = 46.8%, *P* = 0.035; see Fig. 9B). Although the global layer in the mid-region of the CNTF treated muscles had 40.6% fewer slow twitch positive fibers than the naïve control muscles, this difference was not significant (*P* = 0.16). None of the other comparisons were statistically significantly different.

## Discussion

We have shown that short-term treatment of adult rabbit superior rectus muscles with CNTF resulted in decreased force generation for the treated muscles and the muscles contralateral to the treatment compared to naïve controls, but only at the higher stimulation levels. A number of neurotrophic factors have been shown to result in significant alterations in force generation in rabbit and chick extraocular muscles. Several neurotrophic factors and growth factors have showed similar types of effects when directly introduced into specific extraocular muscles, including GDNF, FGF2, and transforming growth factor-β1 (TGF-β1).^11^^,^^20^^,^^21^ The concomitant changes to the superior rectus muscles contralateral to the treatment are common after unilateral treatments, but over the long-term are often no longer present.^12^^–^^14^ Duration of treatment appears to play an important role in changes on the untreated muscle on the contralateral side.

It should be noted that a number of other signaling molecules result in decreased force generation, to include Sonic hedgehog (Shh), Wnt3A, and bone morphogenetic protein 4 (BMP4).^20^^,^^22^ Other studies demonstrated decreased extraocular muscle force generation using antibodies to other neurotrophic and growth factors that were previously shown to increase force, to include antibodies to insulin-like growth factor.^23^ Evidence suggested that alterations in CNTF levels might play a role in the development and/or maintenance of strabismus, where studies suggested it is differentially expressed in the extraocular muscles from individuals with strabismus.^18^^,^^24^ This also implies that alterations in neurotrophic levels may be used to treat strabismus effectively and improve patients’ quality of life in a way that is less invasive, with the potential ability to titrate doses similar to the use of botulinum toxin. This could result in improved outcomes compared to standard strabismus surgery or when used in combination with standard strabismus surgery.

While a good deal is understood about the role of CNTF as a neurotrophic factor in a wide array of neuronal types, including motor neurons,^19^ less is known about its function in skeletal muscle and even less about its function in the extraocular muscles. Only three previous studies have looked at CNTF in extraocular muscles. The first two studies showed its reduction in strabismic muscles.^17^^,^^18^ The third tested effects of CNTF on muscle samples from individuals with paralytic strabismus, but showed opposite effects in superior and inferior oblique muscles.^24^ These contrasting results suggest that its role is pleiotropic, depending on the type of strabismus. Several studies demonstrated that CNTF and the CNTF receptor α (CNTFRα) are expressed in limb skeletal muscle,^25^^,^^26^ and these levels change in response to denervation in hindlimb muscle.^27^^,^^28^ Intramuscular treatment with CNTF alleviated symptoms of limb muscle weakness in mouse models of amyotrophic lateral sclerosis.^29^ Additionally, abolition of CNTF expression resulted in progressive atrophy of the motor neurons and significant reduction in muscle strength.^30^ Due to the known role of CNTF as a neurotrophic factor, the results of both these studies may be describing secondary effects due to altering motor neuron survival or firing properties rather than a direct muscle effect.^29^^,^^30^ CNTFRα also was shown to have an apparent trophic effect on skeletal muscle, but was largely active in developing muscle only.^27^ CNTF and CNTFR are both expressed on neuromuscular junctions, further supporting the view that these effects are likely to have a neuronal component,^31^ as it was upregulated in denervated muscle.^27^ Further experiments are needed to resolve these issues. Thus, it is not altogether surprising that exogenously added CNTF altered muscle force generation capacity, but the mechanism is likely to be more complex than a primary effect on muscle.

The alterations in contractile properties were significant compared to those seen in naïve controls, with the majority of the changes resulting in shortened contraction times and shortened relaxation times. We have seen alterations in contractile properties in adult rabbit extraocular muscle after GDNF treatment^21^ and after brain derived neurotrophic factor treatment (BDNF).^32^ Significant changes in contractile kinetics also were seen in chick extraocular muscle after treatment with a number of different growth factors, such as IGF1 or cardiotrophin-1, as well as after treatment with neutralizing antibodies or binding proteins.^23^ These manipulations demonstrate a wide range in potential ability to alter contractile kinetics in the extraocular muscles, which in the future may potentially be used to treat eye movement disorders such as strabismus and nystagmus.

Cross-sectional myofiber areas were significantly decreased in the orbital and global layers of all CNTF-treated superior rectus muscles. While muscle size is not necessarily linked to muscle force generation capacity,^33^ these results correlated with the decreased force generation with CNTF treatment. Modulation of the myofiber cross-sectional area has been seen in a number of studies using direct short-term treatment of growth and neurotrophic factors to modulate extraocular muscle force in chicks^10^^,^^23^ and rabbits,^9^^,^^11^^,^^20^^,^^34^ as well as in sustained treatment in rabbits^22^^,^^35^ and nonhuman primates.^12^^–^^14^ The CNTF treatment also resulted in a decrease in the percentage of myofibers that expressed the slow twitch MyHC (*myh7*). These modulations in patterns of MyHC expression were seen in other studies using growth and neurotrophic factors in an attempt to modulate extraocular muscle force generation. This change in MyHC composition would be predicted to result in altered shortening velocity,^36^^–^^38^ which is what was seen. These data collectively demonstrate that contractile properties of extraocular muscles can be manipulated with growth factors to alter eye movements, with different factors having significantly different effects.^8^^,^^23^

A number of studies have examined various contractile properties of extraocular muscles in normal human individuals^39^ as well as in individuals with different forms of strabismus during strabismus surgery.^40^^–^^44^ These studies have demonstrated linear force development, and interestingly medial rectus muscles from individuals with esotropia had significantly greater active force, as did the lateral rectus muscles from individuals with exotropia, but not in cases where the strabismus was intermittent.^40^ These data suggest that altering forces in the “too strong” or “too weak” muscles would serve to counteract these differences. Another important finding from these studies is that in measurements with the muscles attached to the sclera or detached from the globe, forces were not significantly different.^44^ This examination of forces in human extraocular muscles provides support that the in vitro force measurements performed in these studies are reflective of what they would be in vivo.

In summary, 3 days of direct CNTF treatment of superior rectus muscles in adult rabbits caused a significant decrease in force generation, albeit only at the higher stimulation frequencies, faster contractile properties to include faster relaxation, and faster contraction times, decreases to myofiber cross-sectional areas of the treated muscles, and a decrease in the number of myofibers expressing slow twitch MyHC. These studies suggest that they have the potential to be used to modulate muscle properties and force generation in strabismic individuals. Future studies are directed toward an approach that would be both sustained, for at least 3 months, and injectable. It might be most efficacious to use any particular neurotrophic factor, such as CNTF here, in tandem with other growth and neurotrophic factors, which have been shown to modulate extraocular muscle contractile properties and force generation parameters, such as IGF1.
